# Draft genome sequence of multidrug-resistant *Bacillus subtilis* strain Hakim RU_LJ isolated from lacchi juice in Bangladesh

**DOI:** 10.1128/mra.00278-25

**Published:** 2025-05-27

**Authors:** Muhib Ullah Khan, Md. Arif-Uz-Zaman Polash, Nusrat Zahan, Md. Mottaleb Ali, Nilima Rubaba Azad, Subir Sarker, Md. Hakimul Haque

**Affiliations:** 1 Department of Veterinary and Animal Sciences, University of Rajshahi 118869, Rajshahi, Rajshahi Division, Bangladesh; 2 Biomedical Sciences & Molecular Biology, College of Medicine and Dentistry, James Cook University 8001, Townsville, Queensland, Australia; 3 Australian Institute of Tropical Health and Medicine, James Cook University 8001, Townsville, Queensland, Australia; Queens College Department of Biology, Queens, New York, USA

**Keywords:** whole genome, lacchi juice, multidrug-resistant, *Bacillus subtilis*, Bangladesh

## Abstract

This report details the draft genome sequence of the multidrug-resistant *Bacillus subtilis* strain Hakim RU_LJ derived from lacchi juice. The assembled genome spans 4.03 Mb, with 50.33× coverage and a GC content of 43.5%. Analysis revealed six CRISPR arrays, 19 prophages, 17 antibiotic resistance genes, and 10 virulence factor genes.

## ANNOUNCEMENT

*Bacillus subtilis*, an aerobic, endospore-forming, Gram-positive bacterium with Generally Recognized as Safe (GRAS) status, can contaminate fermented beverages, such as lacchi juice, through soil, water, or raw ingredients (1–3). Its ability to thrive during fermentation is attributed to its spore-forming nature and adaptability. The bacterium’s multidrug resistance is likely driven by environmental exposure to antibiotics, which facilitates resistance development through mutations or horizontal gene transfer (4). Continuous antimicrobial resistance (AMR) surveillance is crucial for assessing potential risks, enhancing food safety measures, and unlocking biotechnological applications (5, 6).

In May 2024, with authorization from the Institute of Biological Sciences (IBScs) at the University of Rajshahi, Bangladesh (Memo No. 56/321/IAMEBBC/IBScs), we collected samples of lacchi juice from street vendors near the university (24.3733°N, 88.6049°E) using standard protocols. The samples were thoroughly mixed, transferred into sterile containers, and promptly delivered to the laboratory (7). *Bacillus subtilis* was isolated by culturing the samples on nutrient agar (HiMedia, India) and blood agar (HiMedia, India), with aerobic incubation at 37°C for 18–24 hours, followed by microscopic staining and biochemical analyses (8). The antimicrobial resistance patterns of the isolates were evaluated against 10 widely used antibiotics through the disk diffusion technique (9), adhering to CLSI standards (10). A multidrug-resistant strain, showing resistance to tetracycline, doxycycline, cefixime, ceftazidime, and nalidixic acid, was selected for genomic analysis.

The bacterial strain was grown overnight in nutrient broth (HiMedia, India) at 37°C, and its genomic DNA was isolated using the Qiagen DNA Mini Kit (QIAGEN, Hilden, Germany). The extracted DNA was fragmented enzymatically with the NEBNext dsDNA Fragmentase Kit (NEB, MA, USA), and fragments were size-selected using SPRI beads (11)). A sequencing library was constructed with the Nextera DNA Flex Library Preparation Kit (Illumina, San Diego, CA, USA) and sequenced on the Illumina NextSeq2000 platform, generating 2 × 150 paired-end reads. Sequence quality was assessed with FastQC v0.11.7 (12), and the raw reads (*n* = 1,382,869) were processed using Trimmomatic v0.39 (13) to remove low-quality bases and adapters. Genome assembly was carried out with Unicycler v0.4.9 (14), followed by gene annotation using PGAP v3.0 (15). Additional analyses involved screening for antibiotic resistance genes with CARD v3.2.4 (16) and RGI v6.0.2 (17), identifying virulence factors using VFDB and VFanalyzer v4.0 (18), estimating pathogenicity with PathogenFinder v1.1 (19), determining sequence type via MLST v2.0 (20), detecting CRISPR arrays with CRISPRimmunity (21), and evaluating metabolic capabilities with RAST v2.0 (22). Default settings were applied for all software unless specified otherwise.

**Fig 1:**
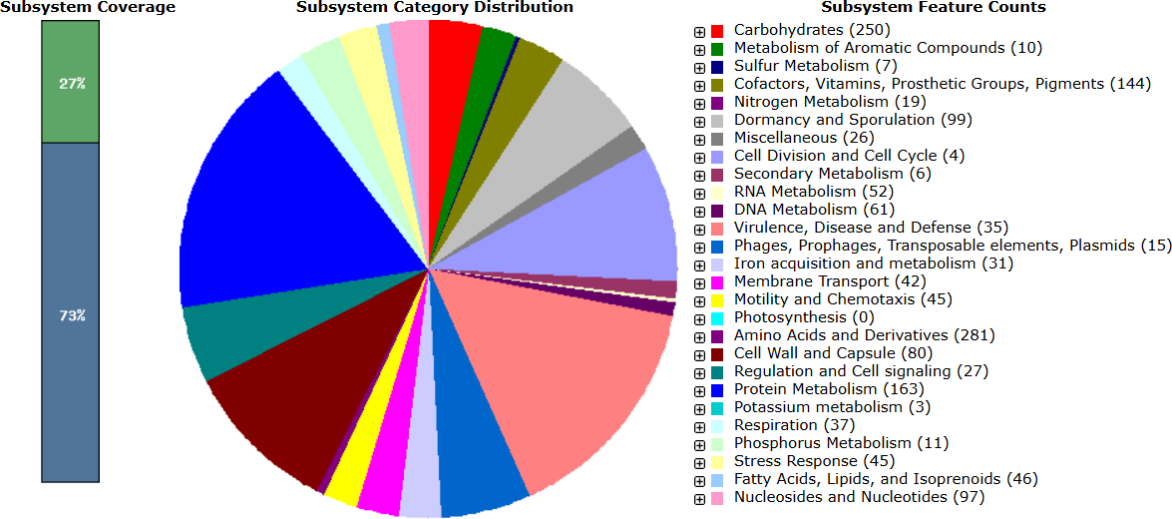
Metabolic functional features in the *Bacillus subtilis* Hakim RU_LJ assembled genome in SEED viewer. The 27% coverage indicates the completeness of functional roles within a specific subsystem across different genomes.

The elements of the draft genomes are documented in Table 1. Notably, 17 ARGs, including putative aadK, mph(K), tet(L), acquired resistance genes and aminoglycosides, macrolide, tetracycline antibiotic classes, and 10 virulence genes comprising ClpP, cap8D, cpsJ, LPG_RS03745, hasC, cap8P, cps4I, wbpM, fepC, and galF were predicted. MLST classified the genome as an unknown sequence type, and the PathogenFinder tool specified a pathogenicity index of 0.236. The genome exhibited five CRISPR arrays with six signature genes (cas3, DEDDh, DinG, TnsC, csa3, and WYL) and 19 prophages. RAST analysis revealed 326 subsystems comprising 4,428 genes with 27% coverage (Fig. 1).

**TABLE 1:** Genomic elements of the *Bacillus subtilis* strain Hakim RU_LJ

Elements	Values
Genome size	4,033,419 bp
Genome coverage	50.33×
G + C content	43.5%
Number of contigs	104
Contig L50	12
Contig N50	110,116 bp
Total genes	4,321
Coding sequences	4,293
Coding genes	4,117
RNA genes	28
tRNAs genes	18
rRNAs genes	4
tmRNAs gene	1
ncRNAs genes	5
Pseudo genes	176
Genes assigned to SEED subsystems	4,428
Number of subsystems	326

## Data Availability

The study on *Bacillus subtilis* strain Hakim RU_LJ, conducted using the WGS shotgun approach, was submitted to NCBI/GenBank, and the assembly was deposited under the accession number JBLYQJ000000000. The pertinent data, including the original readings, were stored with BioProject accession number PRJNA1222092, BioSample accession number SAMN46761393, and SRA accession number SRR32305481. The specific version mentioned in this document is labeled as JBLYQJ000000000.1.
